# Multifunction Sr, Co and F co-doped microporous coating on titanium of antibacterial, angiogenic and osteogenic activities

**DOI:** 10.1038/srep29069

**Published:** 2016-06-29

**Authors:** Jianhong Zhou, Lingzhou Zhao

**Affiliations:** 1 Institute of Physics & Optoelectronics Technology, Baoji University of Arts and Sciences, Baoji, 721016, China; 2 State Key Laboratory of Military Stomatology, Department of Periodontology, School of Stomatology, The Fourth Military Medical University, Xi’an, 710032, China

## Abstract

Advanced multifunction titanium (Ti) based bone implant with antibacterial, angiogenic and osteogenic activities is stringently needed in clinic, which may be accomplished via incorporation of proper inorganic bioactive elements. In this work, microporous TiO_2_/calcium-phosphate coating on Ti doped with strontium, cobalt and fluorine (SCF-TiCP) was developed, which had a hierarchical micro/nano-structure with a microporous structure evenly covered with nano-grains. SCF-TiCP greatly inhibited the colonization and growth of both gram-positive and gram-negative bacteria. No cytotoxicity appeared for SCF-TiCP. Furthermore, SCF-TiCP stimulated the expression of key angiogenic factors in rat bone marrow stem cells (MSCs) and dramatically enhanced MSC osteogenic differentiation. The *in vivo* animal test displayed that SCF-TiCP induced more new bone and tighter implant/bone bonding. In conclusion, multifunction SCF-TiCP of antibacterial, angiogenic and osteogenic activities is a promising orthopedic and dental Ti implant coating for improved clinical performance.

Titanium (Ti) has been widely applied for producing bone implant devices such as joint prostheses, fracture fixation devices and dental implants on account of its excellent biocompatibility and high corrosion resistance^1^,^2^. Nonetheless, Ti itself has no antibacterial activity, on which bacteria can adhere and colonize to form biofilms that are tenacious and highly resistant to antimicrobial treatment, usually resulting in persistent infections and implant failure^3^. Rendering the implants antibacterial to be able to prohibit biofilm formation on it via killing the initially adherent bacteria is believed to be an effective measure to combat the implant related infections^4^. Another aspect needing augmentation for the Ti implants is bone integration. The basis for the long-term normal function of bone implants is rigid bone integration with an intimate bone/implant surface contact. Though a high success rate of nearly 95% has been achieved for the Ti bone implants in healthy patients^5^, they still display deficient bioactivity that may show compromised lifespan and occasional implant failure, especially when encountering some complicated conditions that do not favor the osseointegration establishment, such as osteoporosis. It is generally believed that mesenchymal stem cells (MSCs) play a key role in the implant/bone integration. Most of the osteoblastic cells that colonize the implant surface to form bone originate from MSCs^6^. To accomplish good osseointegration, it is critical for an implant to be able to promote the differentiation of MSCs preferentially towards osteoblasts. Besides, it is more and more recognized that an ideal bone integration process also requires good neovascularization which not only provides nutrition supply for new bone formation but facilitates the distant homing of MSCs to the implant surface for bone formation^7^. Actually, good vascularization also benefits the infection prevention. Growth factors, such as vascular endothelial growth factor (VEGF), are commonly chosen for angiogenesis improvement^8^. However, the high cost as well as the limited functional duration hinders their clinical application^9^.

For advanced multifunction implant coating with the desired antibacterial, angiogenic and osteogenic activities, loading and delivering of inorganic bioactive elements shall be a good strategy. The inorganic elements are quite stable facilitating the incorporation process. Meanwhile, they usually function at very low doses, so relatively long-term effects from the Ti implant of very limited reservoir can be anticipated by properly increasing the loading amounts and controlling the release rate^10^. Regarding the antibacterial elements, silver (Ag) and copper (Cu) exhibit only antibacterial ability, and over-incorporation of them may lead to cytotoxicity^11^,^12^. Different from Ag and Cu, Fluorine (F) has not only excellent antibacterial properties against numerous bacteria but also osteoblastic activity^13^,^14^. Accordingly, F incorporation has drawn considerable attention to enhance the antibacterial activity of medical devices. In terms of the bone formation enhancement, strontium (Sr) has aroused tremendous clinical interests. Sr is reported to increase osteoblast replication, differentiation and bone matrix mineralization as well as to direct the MSC commitment to the bone lineage^15^,^16^. It is noteworthy that F that is mentioned before as an antibacterial element is also an important trace element in human bone and plays a significant role in stimulating osteoblastic activity^14^. For angiogenesis, cobalt (Co) is found to up-regulate the hypoxia-inducible factor (HIF) in cells and in turn promotes angiogenesis by creating a hypoxia mimicking condition. Co incorporated mesoporous bioactive glass and calcium phosphate powder were reported, which had the effect of hypoxia mimicry and showed improved angiogenic property^17^,^18^. It is noted that an excessively high dose of such inorganic elements will induce toxicity^17^, so for their incorporation to the implant surface, an optimal safe dose is important.

Surface modification is a good way to improve the implant biological performance^19^,^20^. Several methods have been applied in synthesizing coatings on the surface of metallic substrates, including plasma spraying^21^, ion implantation^22^, sol-gel precipitation^23^, and so on. However, the coatings formed by these techniques all present some drawbacks, such as low fatigue strength, degradation and a weak adherence to the metallic substrate, as described elsewhere in detail^24^,^25^. Different from the above-mentioned methods, microarc oxidation (MAO), which is an electrochemical method that can form a rough and firmly adherent oxide ceramic layer on certain metals including Ti^26^,^27^,^28^, has been developed as an alternative of metal surface modification. During the MAO process, sparks appear and move rapidly across the treated surface, and the temperature and pressure inside a discharge channel can reach 10^3^–10^4^ K and 10^2^–10^3^ MPa, respectively, which are sufficiently high for inducing an arc thermochemical interaction between the substrate and electrolyte^29^. MAO has been widely investigated to modify the Ti implants for enhanced bioactivity^26^,^27^,^28^,^29^,^30^, and some have already gone to the clinical stage. Meanwhile, MAO also provides an effective means to incorporate the inorganic elements such as calcium (Ca), phosphorus (P), and Sr into the coating via adding them to the electrolyte^27^,^30^. In the present study, four different coatings, namely TiO_2_/Ca-P coating (TiCP), TiCP incorporated with Sr (S-TiCP), TiCP incorporated with Sr and Co (SC-TiCP), and TiCP incorporated with Sr, Co and F (SCF-TiCP), were formed by MAO on Ti. MSCs were seeded onto them to investigate whether they possess favorable cytocompatibility, and angiogenic and osteogenic activities. The antibacterial activity of the coatings against both *Staphylococcus aureus* (*S. aureus*) and *Escherichia coli* (*E. coli*) was examined. Finally, the *in vivo* osseointegration of the coatings was observed in rabbits.

## Results

### Characterization of coatings

**Figure 1:**
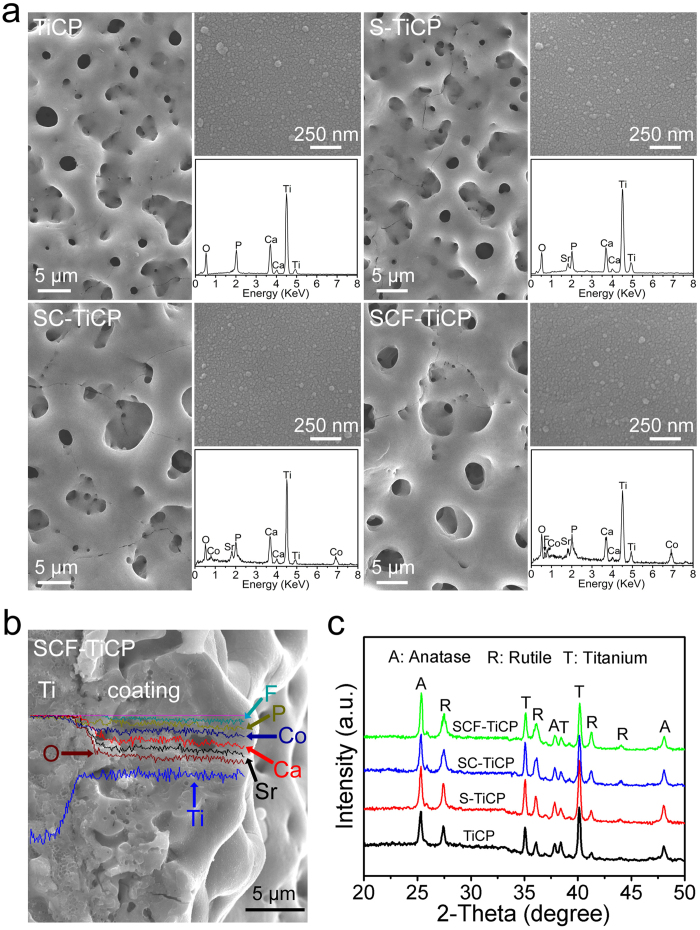
(**a**) SEM surface morphologies of TiCP, S-TiCP, SC-TiCP, and SCF-TiCP, the top insets showing the magnified images and the bottom insets showing the EDX pattern, (**b**) Cross-sectional image of SCF-TiCP and the elements distribution, (**c**) XRD patterns of the coatings.

Figure 1a shows the surface morphology of TiCP, S-TiCP, SC-TiCP, and SCF-TiCP. It can be seen that all coatings are microporous, with pores of an average diameter of 3–4 μm distributing homogeneously over the coatings. The high magnification views further show that all the coatings are fully and evenly covered with nano-grains of ~30–60 nm in size (top insets in Fig. 1a). The energy-dispersive x-ray spectrometer (EDX) results (bottom insets in Fig. 1a) show that only Ti, O, Ca, and P were detected on TiCP, while additional Sr, Sr/Co, and Sr/Co/F elements were detected in S-TiCP, SC-TiCP, and SCF-TiCP, respectively. Table S1 summarizes the surface elemental compositions of the coatings detected by X-ray photoelectron spectroscopy (XPS). The elemental distribution on the cross-section of SCF-TiCP (Fig. 1b) shows that the coating contains Ca, P, Sr and F besides the predominant elements of Ti and O. Theses results further confirmed the successful incorporation of Sr, Co, and F in SCF-TiCP. Furthermore, there was no discontinuity at the interface of the coating/Ti substrate (Fig. 1b), exhibiting a firm binding of the coating to the Ti substrate.

The X-ray diffractometer (XRD) patterns of TiCP, S-TiCP, SC-TiCP, and SCF-TiCP are shown in Fig. 1c. All the coatings consisted of predominant anatase TiO_2_ as well as rutile TiO_2_. The incorporation of Sr, Co, and F slightly changed the phase compositions of the TiO_2_ coating. However, similar to previous work^26^, no feature peaks of Sr, Co, or F-containing compounds were detected in any sample from the XRD patterns because of their very low contents.

**Figure 2:**
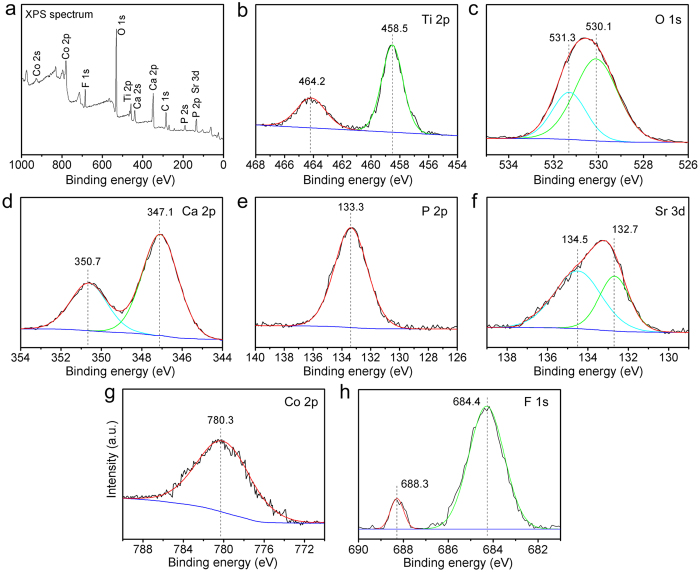
(**a**) XPS survey spectrum, and (**b**) Ti2p, (**c**) O1s, (**d**) Ca2p, (**e**) P2p, (**f**) Sr3d, (**g**) Co2p, and (**h**) F1s high-resolution spectra of SCF-TiCP.

The XPS full spectrum obtained from SCF-TiCP is shown in Fig. 2a. Besides the feature peaks of Ti, O, Ca, and P, feature peaks of Sr, Co, and F were also detected, again confirming the successful incorporation of Sr, Co, and F in SCF-TiCP. The high-resolution spectra of the coating are shown in Fig. 2b–h. The Ti2p spectrum corresponds to typical binding energies for TiO_2_^31^. The O1s spectrum is deconvoluted into two Gaussian component peaks. The peak located at 530.1 eV is assigned to O1s in TiO_2_^31^,^32^, and the other peak at 531.3 eV corresponds to O1s in P = O- groups (Ca_3_(PO_4_)_2_ or CaHPO_4_)^33^,^34^. The Ca2p peaks are located at 347.1 eV and 350.7 eV, and the P2p peak is located at 133.3 eV, which indicates that the Ca2p and P2p exists in the form of calcium phosphate phases (such as α-tricalcium phosphate, and amorphous calcium phosphate) in the detected surface layer^35^, which is a mixture of crystalline and amorphous structures^36^. The doublet peaks of Sr3d are located at around 132.7 eV and 134.5 eV, respectively, in accordance with those reported for SrTiO_3_^37^. The Co2p peak is located at 780.3 eV, assigned to Co2p in CoTiO_3_^38^. The F1s peaks are located at 684.4 eV and 688.3 eV, indicating that a part of the incorporated F exists in the form of Ti-F and the other part goes into the lattice of TiO_2_^39^,^40^.

The roughness values of the coatings measured by atomic force microscopy (AFM) are listed in Table S2. The surface roughness values of the MAO coatings are quite larger compared to that of pristine Ti, however there are no significant differences in the microscale roughness among the MAO coatings, as characterized by the average roughness (Ra), root-mean-square roughness (RMS), and selection of 10-point height of irregularity roughness (Rz). Meanwhile, the contact angles of the water droplets on Ti, TiCP, S-TiCP, SC-TiCP, and SCF-TiCP are 120.6 ± 4.9, 46.9 ± 3.8, 48.2 ± 4.3, 47.6 ± 3.5, and 44.1 ± 5.4°, respectively, indicating that the MAO treatment of Ti can significantly improve its hydrophilicity and the incorporation of Sr, Co, and F in the coatings does not apparently alter the surface wettability of the coating. Similar to previous works^26^, these results further indicate that the incorporation of Sr, Co, and F in the coatings does not apparently influence the surface topography and wettability.

### *In vitro* antibacterial activity

**Figure 3:**
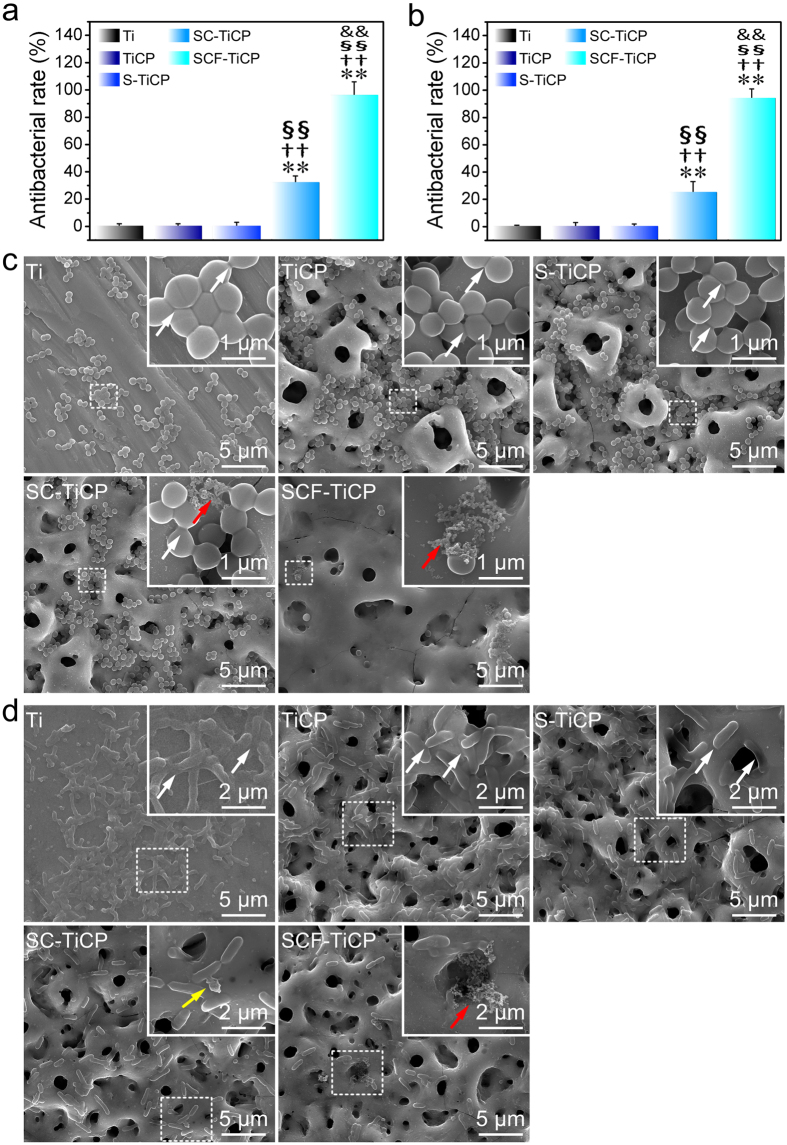
Antibacterial assay against (**a**) *S. aureus* and (**b**) *E. coli*, and SEM images of (**c**) *S. aureus* and (**d**) *E. coli* incubated for 24 h on the coatings. The top insets show the magnified images for the area marked by the square. Data are presented as mean ± SD, n = 4. **p < 0.01 compared to Ti; ^††^*p* < 0.01 compared to TiCP; ^§§^*p* < 0.01 compared to S-TiCP; ^&&^*p* < 0.01 compared to SC-TiCP.

The antibacterial activity of the coatings was examined using both *S. aureus* (Gram-positive bacteria) and *E. coli* (Gram-negative bacteria) colonies. Figure 3a,b show the antibacterial rates for *S. aureus* and *E. coli*, respectively. It can be seen that for both *S. aureus* and *E. coli*, Ti, TiCP, and S-TiCP showed no bactericidal ability with antibacterial rates of nearly 0. On contrary, SC-TiCP and SCF-TiCP surely displayed bactericidal property and SCF-TiCP did better. For SC-TiCP and SCF-TiCP, the antibacterial rates against *S. aureus* were 32.6 ± 1.3% and 97.5 ± 1.8%, respectively, and those against *E. coli* were 25.7 ± 1.6% and 94.8 ± 1.7%, respectively.

Field emission scanning electron microscope (FESEM) examination was performed to investigate the bacterial cell number, morphology and membrane integrity on the Ti surfaces. From Fig. 3c,d, it is observed that there are abundant bacterial cells on Ti, TiCP, S-TiCP and SC-TiCP, while rare cells can be seen on SCF-TiCP. The *S. aureus* cells display intact cell body and smooth surface and on Ti, TiCP, and S-TiCP (as indicated by the white arrows in Fig. 3c). On contrary, on SC-TiCP some cell debris can be seen, and on SCF-TiCP nearly no intact cells are found but some cell debris (as indicated by the red arrows in Fig. 3c). The *E. coli* cells cultured on Ti, TiCP, and S-TiCP are mostly in a rod-shape with abundant binary fission (as indicated by the white arrows in Fig. 3d). On contrary, on SC-TiCP the *E. coli* cells look corrugated and even merged (indicated by the yellow arrow in Fig. 3d), and on SCF-TiCP very little intact cells are found but many completely lysed cells (as indicated by the red arrow in Fig. 3d).

### Protein adsorption, cytotoxicity, cell adhesion, and proliferation

**Figure 4:**
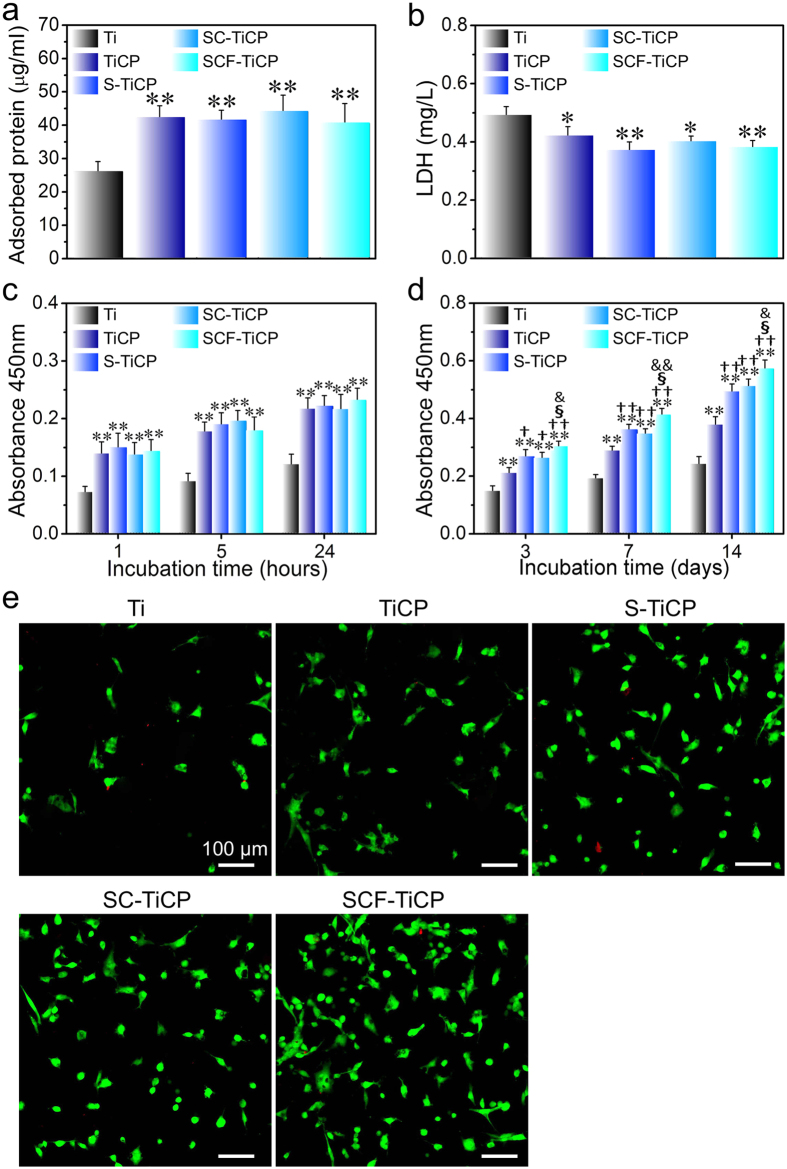
(**a**) Protein adsorption to the samples after 24 h of immersion in a-MEM containing 10% FBS, (**b**) LDH amount released by cells during the first 3 days of incubation, (**c**) cell adhesion measured by CCK-8 after 1, 5, and 24 h of culture, (**d**) cell proliferation measured by CCK-8 after 3, 7, and 14 days of culture, (**e**) Cells incubated for 3 days on the samples stained with two well-described probes, indicating live cells (green) and dead ones (red). Data are presented as mean ± SD, n = 5. *p < 0.05 and **p < 0.01 compared to Ti; ^†^*p* < 0.05 and ^††^*p* < 0.01 compared to TiCP; ^§^*p* < 0.05 compared to S-TiCP; ^&^*p* < 0.05 and ^&&^*p* < 0.01 compared to SC-TiCP.

The protein adsorbed onto a biomaterial is considered to mediate the biological effect of the surface topographical cue to the cells/tissues^41^. Hence, the amount of total protein adsorbed onto the coatings from the cell culture medium after 24 h of incubation was measured (Fig. 4a). In comparison with the pristine Ti control, TiCP, S-TiCP, SC-TiCP, and SCF-TiCP gave rise to more adsorbed proteins, which can be attributed to their rougher surfaces with larger surface areas. TiCP, S-TiCP, SC-TiCP, and SCF-TiCP did not induce obvious difference in the adsorbed protein amount, indicating that the incorporated inorganic elements have no obvious effect on this aspect.

Since Sr, Co, and F overdose may lead to cytotoxicity^17^,^42^, the lactate dehydrogenase (LDH) released by cells cultured on the samples was evaluated as an indication of cytotoxicity. As shown in Fig. 4b, TiCP, S-TiCP, SC-TiCP, and SCF-TiCP exhibited no cytotoxicity compared to the Ti control. Hence, the Sr, Co, and F amounts released from all the samples are considered to be safe. Interestingly, the LDH release induced by TiCP, S-TiCP, SC-TiCP, and SCF-TiCP was slightly smaller than that induced by the Ti control, indicating even enhanced cytocompatibility for the MAO coating compared to the pristine Ti, which is further supported by the subsequent *in vitro* cell adhesion and proliferation data.

From the fluorescent cell viability staining images in Fig. 4e, nearly no dead cells were observed on all the Ti samples after culturing for 3 days, confirming the good cytocompatibility of the coatings. Initial cell adhesion is a key step for the ensuing cell proliferation and differentiation on biomaterials^43^. As shown in Fig. 4c, TiCP, S-TiCP, SC-TiCP, and SCF-TiCP led to more initial adherent cells in comparison with the pristine Ti control, in accordance with their larger surface area and more protein adsorption. However, they did not show obvious difference in the initial adherent cell number among themselves, indicating that the Sr, Co, and F incorporation has a negligible effect on the initial adherent cell number. All the Ti surfaces can well support cell growth as the cells proliferated with time on them all (Fig. 4d). The cell proliferation on TiCP, S-TiCP, SC-TiCP, and SCF-TiCP was obviously better than that on the Ti control, which showed the general trend of SCF-TiCP > SC-TiCP ≈ S-TiCP > TiCP > Ti, and SCF-TiCP had the strongest ability in promoting cell proliferation.

### Cell morphology

**Figure 5:**
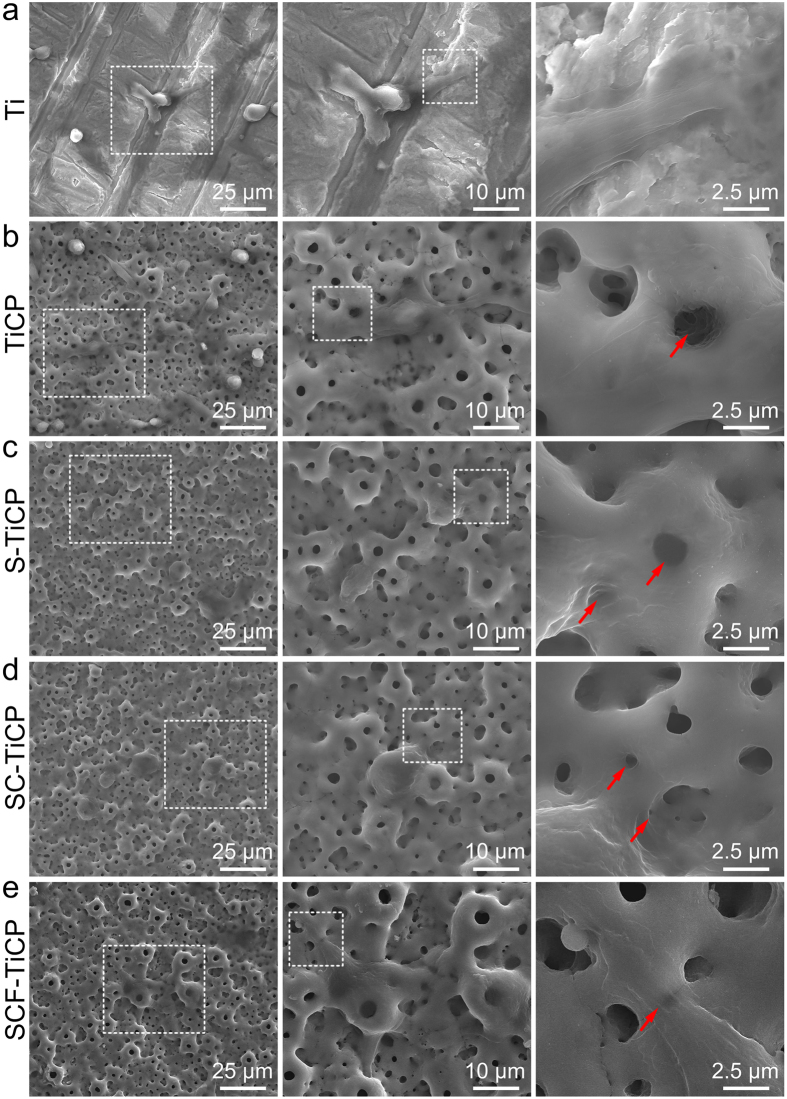
Morphology of MSCs after 3 days of culture on different samples: (**a**) Ti, (**b**) TiCP, (**c**) S-TiCP, (**d**) SC-TiCP, and (**e**) SCF-TiCP.

The cell shape on a biomaterial surface is closely related to the cell functions^44^. In order to observe cell adhesion and spreading, MSCs cultured on different Ti surfaces were examined by FESEM after 3 days of culture (Fig. 5). MSCs on the flat Ti spread poorly with a spindle shape indicative of undifferentiated quiescent cells. Whereas, TiCP, S-TiCP, SC-TiCP, and SCF-TiCP improved the MSC attachment and rendered them spread out extensively, which covered or anchored to the micropores on the surfaces, as indicated by the red arrows in Fig. 5. It can be inferred that the incorporation of Sr, Co, and F does not obviously influence the initial adhesion and spreading of MSCs in comparison with the TiCP group.

### Angiogenesis potential and osteogenesis activity

**Figure 6:**
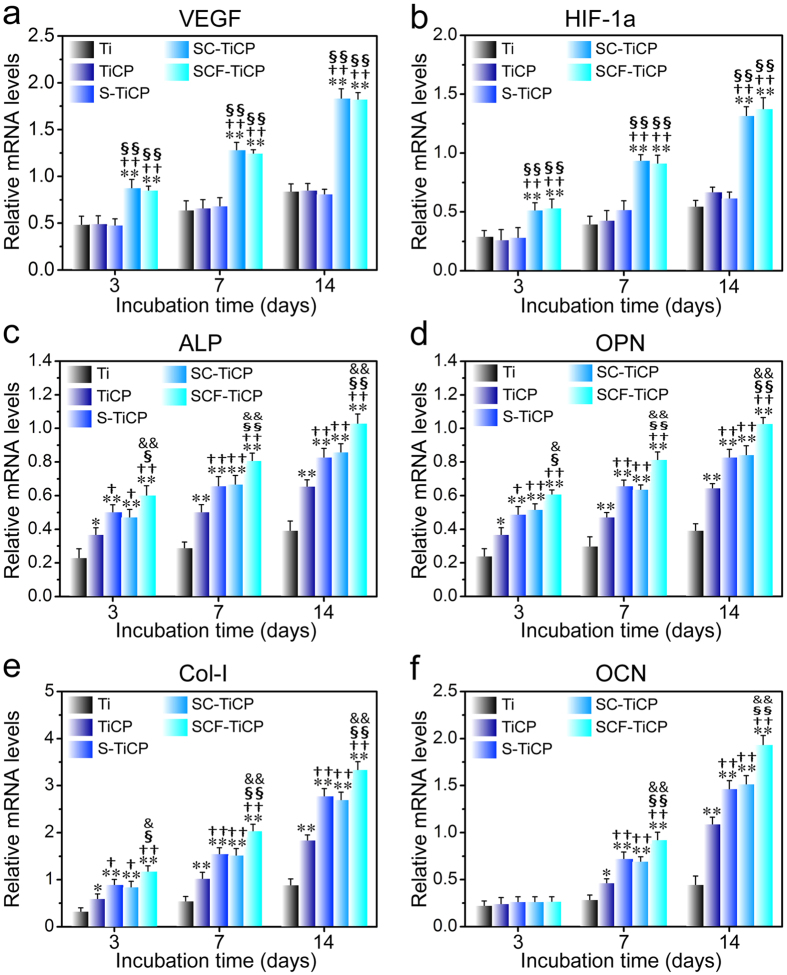
Gene expression of MSCs cultured on the coatings for 3, 7, and 14 days: (**a**) VEGF, (**b**) HIF-1a, (**c**) ALP, (**d**) OPN, (**e**) Col-1, and (**f**) OCN. All values are normalized to GAPDH. Data are presented as mean ± SD. *p < 0.05 and **p < 0.01 compared to Ti; ^†^*p* < 0.05 and ^††^*p* < 0.01 compared to TiCP; ^§^*p* < 0.05 and ^§§^*p* < 0.01 compared to S-TiCP; ^&^*p* < 0.05 and ^&&^*p* < 0.01 compared to SC-TiCP.

**Figure 7:**
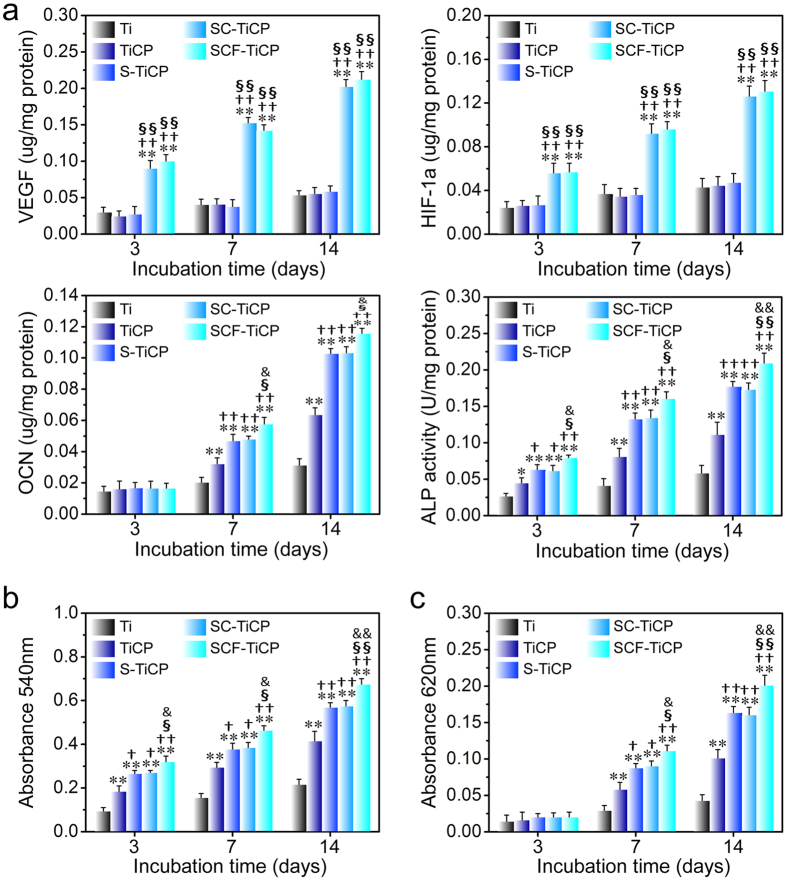
(**a**) Intracellular protein contents of VEGF, HIF-1a, and OCN as well as ALP activity, (**b**) collagen secretion, and (**c**) ECM mineralization by MSCs after 3, 7, and 14 days of incubation on the coatings. Data are presented as mean ± SD, n = 5. *p < 0.05 and **p < 0.01 compared to Ti; ^†^*p* < 0.05 and ^††^*p* < 0.01 compared to TiCP; ^§^*p* < 0.05 and ^§§^*p* < 0.01 compared to S-TiCP; ^&^*p* < 0.05 and ^&&^*p* < 0.01 compared to SC-TiCP.

Quantitative real-time PCR assay was performed to detect the gene expression of key angiogenic factors (VEGF and (hypoxia-inducible factor-1a (HIF-1a)) and the osteogenic factors (alkaline phosphatase (ALP), osteopontin (OPN), type 1 collagen (Col-I), and osteocalcin (OCN)) in MSCs after culturing on various Ti samples for 3, 7, and 14 days (Fig. 6). Meanwhile, the intracellular ALP activity, protein contents of VEGF, HIF-1a, and OCN, collagen secretion and extracelluar matrix (ECM) mineralization were also measured (Fig. 7). Figures 6a,b and 7a show that the VEGF and HIF-1a gene expression and protein product in MSCs on the Ti surfaces followed the rank of SCF-TiCP ≈ SC-TiCP > S-TiCP ≈ TiCP ≈ Ti, indicating that the Co incorporation remarkably increased the expression of angiogenic factors compared to the Co free surfaces. Meanwhile, the gene expression of osteogenic factors (ALP, OPN, Col-I, and OCN), the intracellular ALP activity, protein content of OCN, collagen secretion and ECM mineralization (Figs 6c–f and 7a–c) generally followed the rank of SCF-TiCP > SC-TiCP ≈ S-TiCP > TiCP > Ti, indicating that the Sr and F incorporation significantly enhanced the osteogenic differentiation of MSCs while the Co incorporation seemed not.

### Bone formation *in vivo*

**Figure 8:**
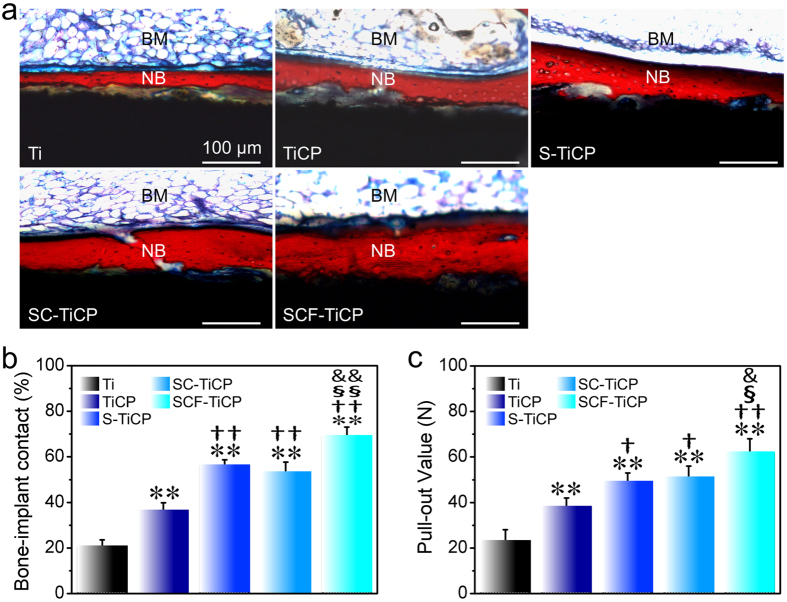
(**a**) Histological analysis of the implant/bone interface after implantation in the rabbit model for 8 weeks. The tissues stained in red are the newly formed bone, and the tissues stained in yellow are the fibrous tissue. NB indicates the newly formed bone. BM indicates the bone marrow cavity. (**b**) Percentage of BIC and (**c**) the pull-out values after 8 weeks of implantation. Data are presented as mean ± SD, n = 3. **p < 0.01 compared to Ti; ^†^*p* < 0.05 and ^††^*p* < 0.01 compared to TiCP; ^§^*p* < 0.05 and ^§§^*p* < 0.01 compared to S-TiCP; ^&^*p* < 0.05 and ^&&^*p* < 0.01 compared to SC-TiCP.

Finally, the *in vivo* bone formation of the coatings was assessed in rabbits after 6 weeks of healing. The bone/implant interface was histologically inspected by Van Gieson’s staining (Fig. 8a). All the Ti surfaces induced new bone deposition but of different amounts, following the obvious trend of SCF-TiCP > SC-TiCP ≈ S-TiCP > TiCP > Ti. On Ti and TiCP (especially Ti), the new bone is separated from the Ti sample surfaces by a thin layer of fibrous connective tissue (yellow color). However, on S-TiCP, SC-TiCP, and SCF-TiCP, the new bone bonds tightly to the implant surface without discernible fibrous intervening. The percentage of BIC calculated based on the Van Gieson’s staining (Fig. 8b) and the biomechanical strength of bone-implant integration measured by pull-out test (Fig. 8c) also followed the rank of SCF-TiCP > SC-TiCP ≈ S-TiCP > TiCP > Ti. Totally, the *in vivo* bone integration ability of the coatings is well in line with their *in vitro* effect on MSC functions. SCF-TiCP has the strongest ability to establish *in vivo* osseointegration.

## Discussion

The advanced multifunction coating with the antibacterial, angiogenic and osteogenic activities is required for improved clinical performance of Ti implant, and we propose that co-doping and delivering of inorganic bioactive elements including Sr, Co, and F is a good strategy. Birgani *et al*. recently investigated the potential of using a combination of F and Co ions by incorporating them into the calcium phosphate to simultaneously promote osteogenesis and angiogenesis in MSCs. Their results revealed that the Co ions increased the expression of angiogenic markers and the F ions, individually or in combination with Co, significantly increased the expression of many of the selected osteogenic markers, as well as mineral deposition^45^. Birgani *et al*.’s study is well in line with our idea. It is widely accepted that all the surface properties of a biomaterial including surface chemistry, surface energy/wettability, surface roughness and topography can influence its biological performance^43^. The different MAO Ti coatings fabricated in this study have the same microporous feature and phase composition of TiO_2_, and similar surface roughness and wettability. Therefore, one can assess the effects of Sr, Co, or F incorporation on the biological performances of the coating without involving the other factors.

Even though Sr, Co, and F have been previously proposed as dopants to improve the performance of biomaterials, the feasibility of their co-incorporation, specifically into the TiO_2_ coating, is unclear. Our present study demonstrates that MAO provides a feasible and facile strategy for the co-incorporation of multiple inorganic elements to the Ti implant surface via adding them to the MAO electrolyte. By this approach, Sr, Co, and F can be successfully incorporated, while the typical MAO microporous structure that benefits bone integration can be well retained. Figure S1 shows that the incorporated elements can be released in a relatively slow mode from corresponding samples to exert their biological responses.

SC-TiCP showed certain antibacterial ability against the colonization of *S. aureus* and *E. coli* and SCF-TiCP exhibited excellent antibacterial ability, indicating that the Co and F incorporation could assign the implant with antibacterial ability against both gram-positive and gram-negative bacteria, and the F incorporation possesses higher efficiency than Co in this aspect. F can affect bacterial metabolism via a direct effect of an enzyme inhibitor as well as via forming metal-F complexes that are responsible for inhibition of proton-translocating F-ATPases^46^,^47^. Figure S1 shows that F ions can be released from SCF-TiCP. Furthermore, the XPS spectra in Fig. 2 display that Ti-F complex is formed in the coating. Hence, the antibacterial effect of SCF-TiCP shall be partly ascribed to the F release and the formation of Ti-F complex. Meanwhile, Co also contributes a part, as some researches indicated that the Co incorporation of into metal oxide improved its antibacterial activity^48^,^49^.

Considering the similar surface topography, wettability, microstructure and phase compositions of the coatings, the incorporation of Sr and F is thought to be the reason for the enhanced MSC proliferation by S-TiCP, SC-TiCP, and SCF-TiCP compared to TiCP. Actually, the incorporation of Sr or F into biomaterials was proven to be beneficial to cell proliferation while had no influence on cell adhesion^14^,^50^, and suitable amount of Co incorporated into bioactive glass scaffolds had no significant cytotoxicity^17^, which support our present data. Numerous studies^17^,^18^,^51^ revealed that Co could significantly stimulate new vessel formation. A widely accepted mechanism is that it can mimic the hypoxia condition via stabilizing HIF-1α to activate the VEGF expression that is associated with neovascularization and tissue regeneration. There are some studies indicating that the Co incorporation into biomaterials (bioactive glass or hydroxyapatite) could surely mimic a hypoxia condition, up-regulate HIF-1a and VEGF expression, and thus improve the angiogenic properties of these biomaterials^17^,^52^,^53^. It was reported that Sr affected the behaviors of osteoblasts, osteoclasts, and MSCs to modulate the bone turnover towards bone formation^30^,^32^,^42^,^54^. Previous studies suggested that the Sr incorporation into the MAO TiO_2_ coating exhibited significant enhancement in osteoblast differentiation *in vitro* and new bone formation *in vivo*^30^,^32^. With regard to F, its introduction to the hydroxyapatite coating was widely tried and it was proven that certain dosage of F was beneficial to osteoblast differentiation^14^,^55^. Even though Sr, Co, and F have been proposed as dopants to improve the performance of biomaterials, their biological effect after co-incorporation has never been studied. Here SCF-TiCP simultaneously up-regulated the expression of both angiogenic factors and osteogenic factors from MSCs, suggesting the beneficial co-effect of the Sr, Co, and F on both vascularization and osteogenic differentiation as required.

Finally, the animal test has been conducted to evaluate the *in vivo* osseointegration of coatings. A rat femur implantation model was adopted and the implants were inserted in the femoral shaft where MSCs exist abundantly. The data showed that after 6 weeks the new bone formation, the percentage of bone-to-implant contacts (BIC), and the biomechanical strength of bone-implant integration all followed the rank of SCF-TiCP > SC-TiCP ≈ S-TiCP > TiCP > Ti (Fig. 8), which were well in line with the *in vitro* data on MSCs. It is inferred that the coatings may influence the *in vivo* osseointegration via modulating the functions of MSCs just as observed *in vitro*. SCF-TiCP had the strongest ability to establish *in vivo* osseointegration.

## Methods

### MAO treatment of pure Ti

The pure Ti disks with size of ϕ15 × 2 mm were set as anodes and treated in aqueous electrolytes of different chemical compositions (Table S3) with an applied positive pulse voltage of 410 V, a negative pulse voltage of 100 V, a pulse frequency of 100 Hz, and a duty ratio of 26% for 5 min. The MAO treated Ti samples were ultrasonically washed with alcohol and distilled water, and then dried at room temperature.

### Surface characterization

The morphology and elemental composition of the coatings were examined by FESEM (JEOL JSM-6700F, Japan) equipped with EDX. Phase identification was carried out with XRD (X’Pert PRO, The Netherlands). The elements and chemical species of the coatings were examined with XPS (Axis Ultra, UK). The roughness of the coatings was examined by AFM (SPM-9500J3, Japan). The hydrophilicity of the coatings was measured on a surface contact angle measurement machine (DSA30, Kruss, Germany).

### Determination of ions released

The specimens were immersed in 10 ml α-minimum essential medium (α-MEM) (Life Technologies, USA) at 37 °C for 1, 3, 6, 14, and 28 days successively. At the pre-determined time points, the leaching liquid was collected and the concentrations of Ca, P, Sr, and Co ions released were measured by inductively coupled plasma-mass spectrometry (ICP-MS; Nu Instruments, Wrexham, UK). The F concentrations were measured using a fluoride ion electrode (9409SC, Orion Research, UK) connected to an Ion Analyzer (901, Orion Research, UK), after diluting with an ionic strength adjustment buffer (TISAB with CDTA). The data were normalized to a standard curve obtained with a standard fluoride solution (Fluoride standard, Orion Research, UK) within a range of 0.1–20 ppm. Meanwhile, the Ca, P, Sr, Co, and F ion concentrations in the fresh α-MEM were measured as the background using the above-mentioned methods. The difference of the ion concentrations between the specimen-immersed α-MEM and the fresh α-MEM indicates the concentrations of ion release from coatings. The ion release tests were performed on five replicate samples (n = 5).

### Protein adsorption assay

The protein adsorption assay was conducted in α-MEM containing 10% fetal bovine serum (FBS; Life Technologies, USA). After incubation in the medium for 24 h at 37 °C, the proteins adsorbed onto the samples were detached by 1% sodium dodecyl sulfate (Solarbio) and determined using a MicroBCA protein assay kit (Pierce). Five samples for each group were tested.

### Antibacterial activity evaluation

The antimicrobial effect of the specimens was evaluated by the bacterial counting method with *E. coli* (ATCC10536) as the gram-negative representative and *S. aureus* (ATCC6538) as the gram-positive representative. The detailed process is shown in Supplementary Materials.

### MSC harvest and culture

MSCs were harvested from ten 1-week-old New Zealand rabbits^56^. The method is shown in Supplementary Materials. All experiments were performed within passage 3. MSC suspension of 1 ml with 2 × 10^4^ cells was seeded on the Ti samples.

### Cytotoxicity, cell adhesion, proliferation, and morphology

The activity of LDH in the culture media released by the cells was used as an index of cytotoxicity. After culturing for 3 days, the culture medium was collected (among this period the medium was not changed) and centrifuged, and the LDH activity in the supernatant was determined spectrophotometrically according to the manufacturer’s instruction (Sigma, USA).

The cell counting kit-8 (CCK-8) assay was used to assess the adhesion and proliferation of MSCs after culture of 1 h, 5 h, 24 h, 3 days, 7 days, and 14 days.

Live/dead staining using the LIVE/DEAD Viability/Cytotoxicity Kit (Invitrogen, France) was performed to identify viable and nonviable MSCs on the samples after 3 days of incubation. The cell-adherent samples were incubated with 500 μL of phosphate buffer saline (PBS) containing ethidium-homodimer-1 (4 μM) and calcein-AM (2 μM) at 37 °C for 30 min. The fluorescence-stained cells were analyzed using an OLYMPUS laser confocal microscope (FV1000).

After 3 days of incubation, the samples with MSCs were washed with PBS, fixed in 3% glutaraldehyde, dehydrated in a graded ethanol series, freeze-dried, and sputter-coated with gold prior to observation by the FESEM.

### Quantitative real-time PCR assay

After culturing for 3, 7, and 14 days, the total RNA was isolated using the TRIzol reagent (Life Technologies, USA), and 1 μg RNA from the cells on each sample was reversed transcribed into complementary DNA using a PrimeScrip RT reagent kit (TaKaRa, Japan). The expression of key angiogenic factors (HIF-1a and VEGF) and osteogenic differentiation markers (ALP, OPN, Col-I, and OCN) was quantified on a quantitative real-time polymerase chain reaction (qRT-PCR) detection system (Bio-Rad iQ5 Multicolor) with SYBRPremix ExTaqII (TaKaRa, Japan). Data analysis was carried out using an iQ5 Optical System (Bio-Rad, USA) with software version 2.0. The expression levels of the target genes were normalized to that of the housekeeping gene glyceraldehyde-3-phosphate dehydrogenase (GAPDH). The primers for the target genes were listed in Table S4.

### Intracellular ALP activity and contents of specific proteins

After 3, 7 and 14 days of culture, the cell-seeded samples were washed thrice with PBS, lysed in 0.1 vol% Triton X-100 (Life Technologies, USA) through five standard freeze-thaw cycles, and finally shaken for 10 min. The intracellular ALP activity and intracellular contents of proteins (VEGF, HIF-1a, and OCN) in the cell lysates were determined with ELISA (Bluegene Ltd., China). The optical absorbance at 450 nm was recorded spectrophotometrically, and the ALP activity and the protein contents of MSCs cultured on the samples were drawn from a standard curve of absorbance versus known standards of corresponding proteins run in parallel with the experimental samples. The results were normalized to the intracellular total protein content. Five samples for each group were tested (n = 5).

### Collagen secretion and extracelluar matrix mineralization

Collagen secretion and ECM mineralization on the samples were assessed after 3, 7, and 14 days of culture by the Sirius Red and Alizarin Red staining, respectively. After washing with PBS and fixation, the samples were stained using 0.1% Sirius Red (Sigma, USA) to reveal the collagen and 40 mM Alizarin Red (pH 4.2, Sigma, USA) to show the mineralization. In the quantitative analysis, the Sirius Red or Alizarin Red stain on the specimen after washed with 0.1 M acetic acid or distilled water was dissolved in 0.2 M NaOH/methanol (1:1) or 10% cetylpyridinum chloride (Acros) to measure the optical density at 540 nm or 620 nm.

### Animal experiment

The animal experiment was conducted according to the ISO 10993-2:1992 animal welfare requirements and approved by the Institutional Animal Care and Use Committee (IACUC) of Xi’an Jiaotong University. Twelve adult New Zealand rabbits weighing 2.5–3 kg were used. The rabbits were anesthetized with 0.5% pentobarbital sodium. Ti cylinders (Ф2.5 × 10 mm) with different coatings (n = 3 for each coating) were implanted into the left femoral shaft of the rabbits. After the operation, all rabbits received a prophylactic subcutaneous injection of gentamycin. The animals were sacrificed under general anesthesia 8 weeks after implantation. Then the implants with surrounding tissue were retrieved for examination. The percentage of BIC was assessed on 3 to 4 sections on each implant and all the areas of interest were within the endosseous part of the implants. The images were captured by a fluorescence microscope (Olympus IX 71, Olympus, Japan) and analyzed using PC-based image analysis system (Image-Pro R Analyzer 7.0 Japan). The exact position of the implants inserted in the rabbit femoral shaft and the region for histological analysis are shown in Figure S2.

The biomechanical pull-out test was used to assess the strength of bone-implant integration. The femora containing a cylindrical implant (n = 3 for each group) was harvested after 8 weeks of healing, and two segments of the femora were partially embedded in PMMA with the implant top being horizontal. The testing machine (Shimadzu, AGS-10kNG, Japan) was used to pull the implant vertically out at a cross-head speed of 1 mm/min. The load-displacement curve was recorded and the maximum pull-out force was then calculated.

### Statistical analysis

The data were expressed as mean ± standard deviation (SD) from three independent experiments. The data were analyzed using SPSS 14.0 software (SPSS, USA). A one-way ANOVA followed by a Student-Newman-Keuls *post hoc* test was used to determine the level of significance. *p* < 0.05 and 0.01 was considered to be significant and highly significant, respectively.

## Conclusions

Microporous TiO_2_/Ca-P coating doped with Sr, Co, and F has been successfully developed through a simple MAO procedure. *In vitro* tests demonstrate that SCF-TiCP simultaneously possesses multiple functions of antimicrobial, angiogenic and osteogenic capabilities. *In vivo* experiment indicates that SCF-TiCP induces more new bone formation and tighter bone bonding. To the best of our knowledge, this is the first successful attempt to fabricating the multifunction Sr, Co, and F co-doped microporous TiO_2_/Ca-P coating, being of great potential for dental and orthopedic applications.

## Additional Information

**How to cite this article**: Zhou, J. and Zhao, L. Multifunction Sr, Co and F co-doped microporous coating on titanium of antibacterial, angiogenic and osteogenic activities. *Sci. Rep.* **6**, 29069; doi: 10.1038/srep29069 (2016).

## Supplementary Material

Supplementary Information
