# Supplementary material for: Multifunction Sr, Co and F co-doped microporous coating on titanium of antibacterial, angiogenic and osteogenic activities
Source: Sci Rep. 2016 Jun 29;6:29069. doi: 10.1038/srep29069 (PMC4926257; doi:10.1038/srep29069)
Supplement: Supplementary Information [file srep29069-s1.doc]

**Supplementary Materials**

**Multifunction Sr, Co and F co-doped microporous coating on titanium of** **antibacterial,** **angiogenic and osteogenic activities**

Jianhong Zhou ^1^ and Lingzhou Zhao ^2*^

^1^ Institute of Physics & Optoelectronics Technology, Baoji University of Arts and Sciences, Baoji, 721016, China

^2^ State Key Laboratory of Military Stomatology, Department of Periodontology, School of Stomatology, The Fourth Military Medical University, Xi’an, 710032, China

*^*^Corresponding author:*

*State Key Laboratory of Military Stomatology, Department of Periodontology, School of Stomatology, The Fourth Military Medical University, No. 145 West Changle Road, Xi’an 710032, China. Dr. Lingzhou Zhao, E-mail:* [*zhaolingzhou1983@hotmail.com*](mailto:zhaolingzhou1983@hotmail.com)

**Methods**

**Antibacterial activity evaluation**

The bacteria were grown overnight in Luria–Bertani (LB) medium containing 10 g L^-1^ peptone, 5 g L^-1^ NaCl, and 5 g L^-1^ beef extract. The pH was adjusted to be between 7.0 and 7.2 using 1 M NaOH solution. *S. aureus* and *E. coli* were then re-suspended in phosphate buffered saline (PBS) at a concentration of 10^7^ and 10^6^ cells/ml (calibrated in terms of colony forming units (CFU) using the spread plate method), respectively. One ml of the bacterial suspension discussed above was added to each sample and incubated for 24 h at 37^o^C. Then the bacteria on the samples were dissociated, collected and inoculated into a standard agar culture medium. After incubation at 37^o^C for another 24 h, the live bacteria were counted in accordance with the National Standard of China GB/T 4789.2 protocol. The antibacterial ratio was calculated using the formula, A.R.= (A-B)/A×100%, where A.R. means the antibacterial ratio, A is the average number of bacteria on the control specimen, the flat Ti (CFU per specimen), and B is the average number of bacteria on the testing specimen (CFU per specimen).

In the FESEM examination, 1 ml of the bacterial suspension discussed above was inoculated to each Ti sample and incubated for 24 h at 37^o^C. The samples were fixed, dehydrated in a series of ethanol solutions for 30 min each followed by the final dehydration conducted in absolute ethanol twice, dried in the hexamethyldisilizane ethanol solution series, and finally observed with FESEM.

**MSC harvest and culture**

The animal experiments were conducted according to the ISO 10993-2:1992 animal welfare requirements and approved by the Institutional Animal Care and Use Committee (IACUC) of Xi'an Jiaotong University. Briefly, bone marrow was aspirated from the femora and tibias, from which the mononucleated cells were isolated via density gradient centrifugation. The cells obtained were plated in cell culture flasks containing 20 ml of a-MEM containing 10% FBS and 1% antibiotics, and cultured at 37 ^o^C in a humidified atmosphere of 5% CO_2_ and 95% air. Non-adherent cells were removed and the adherent cells were collected for further expanding.

**Table S1.** Elemental composition of the coatings surfaces detected by XPS.

| Coatings | Elemental composition (wt.%) | | | | | | |
| --- | --- | --- | --- | --- | --- | --- | --- |
|  | Ti | O | Ca | P | Sr | Co | F |
| TiCP | 47.6±0.5 | 42.2±0.4 | 5.9±0.3 | 4.3±0.2 | - | - | - |
| S-TiCP | 43.8±0.6 | 39.6±0.3 | 5.6±0.4 | 4.3±0.1 | 6.7±0.3 | - | - |
| SC-TiCP | 41.0±0.4 | 38.1±0.5 | 5.8±0.5 | 3.9±0.2 | 6.1±0.2 | 5.1±0.3 | - |
| SCF-TiCP | 38.9±0.5 | 36.2±0.4 | 6.0±0.2 | 4.1±0.3 | 6.4±0.4 | 4.9±0.2 | 3.5±0.3 |

**Table S2.** Roughness values and contact angles of pristine Ti and the coatings.

| Coatings | Roughness (nm) | | | Contact angle  (deg.) |
| --- | --- | --- | --- | --- |
|  | Ra | RMS | Rz |  |
| Ti | 6.1±1.2 | 8.2±1.5 | 38.4±4.6 | 120.6±4.9 |
| TiCP | 431.8±42.5 | 459.2±39.4 | 1186.3±138.2 | 46.9±3.8 |
| S-TiCP | 462.7±41.3 | 475.9±35.7 | 1229.8±140.9 | 48.2±4.3 |
| SC-TiCP | 456.4±31.6 | 480.1±30.9 | 1206.2±156.7 | 47.6±3.5 |
| SCF-TiCP | 470.2±38.7 | 483.5±38.1 | 1215.6±149.3 | 44.1±5.4 |

**Table S3.** The corresponding MAO electrolyte compositions.

| Coatings | Aqueous electrolyte concentration (M) | | | | |
| --- | --- | --- | --- | --- | --- |
|  | Calcium acetate | β-glycerophosphate disodium | Strontium acetate | Cobalt acetate | Sodium fluoride |
| TiCP | 0.05 | 0.02 | - | - | - |
| S-TiCP | 0.05 | 0.02 | 0.05 | - | - |
| SC-TiCP | 0.05 | 0.02 | 0.05 | 0.04 | - |
| SCF-TiCP | 0.05 | 0.02 | 0.05 | 0.04 | 0.1 |

**Table S4. Primers used for qRT-PCR.**

| Gene | Forward primer sequence (5′-3′) | Reverse primer sequence (5′-3′) |
| --- | --- | --- |
| VEGF | TTGAGTTGGGAGGAGGATGT | TGGCAGGCAAACAGACTTC |
| HIF-1a | CGATGACACGGAAACTGAAG | CAGAGGCAGGTAATGGAGACA |
| ALP | CTGAGCGTCCTGTTCTGAGG | GTTCCTGGGTCCCCTTTCTG |
| OPN | GTGTACCCCACTGAGGATGC | CACGTGTGAGCTGAGGTCTT |
| OCN | CTTCGTGTCCAAGAGGGAGC | CAGGGGATCCGGGTAAGGA |
| Col-I | TGCAGGGCTCCAATGATGTT | TGCAGGGCTCCAATGATGTT |
| GAPDH | ATCAAGTGGGGTGATGCTGG | TACTTCTCGTGGTTCACGCC |


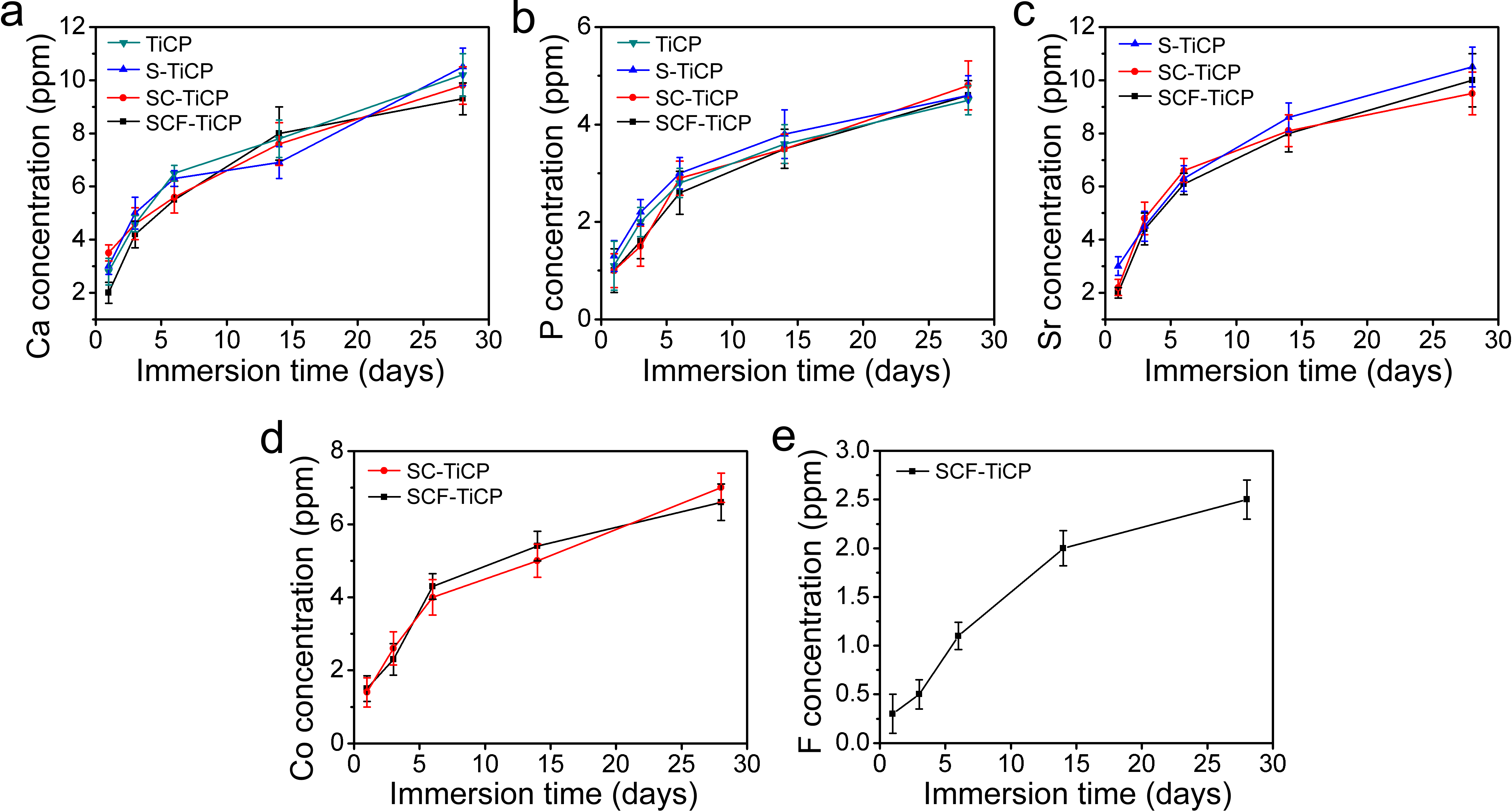


**Figure S1.** Cumulative release of (a) Ca, (b) P, (c) Sr, (d) Co, and (e) F by TiCP, S-TiCP, SC-TiCP, and SCF-TiCP for various days.

**
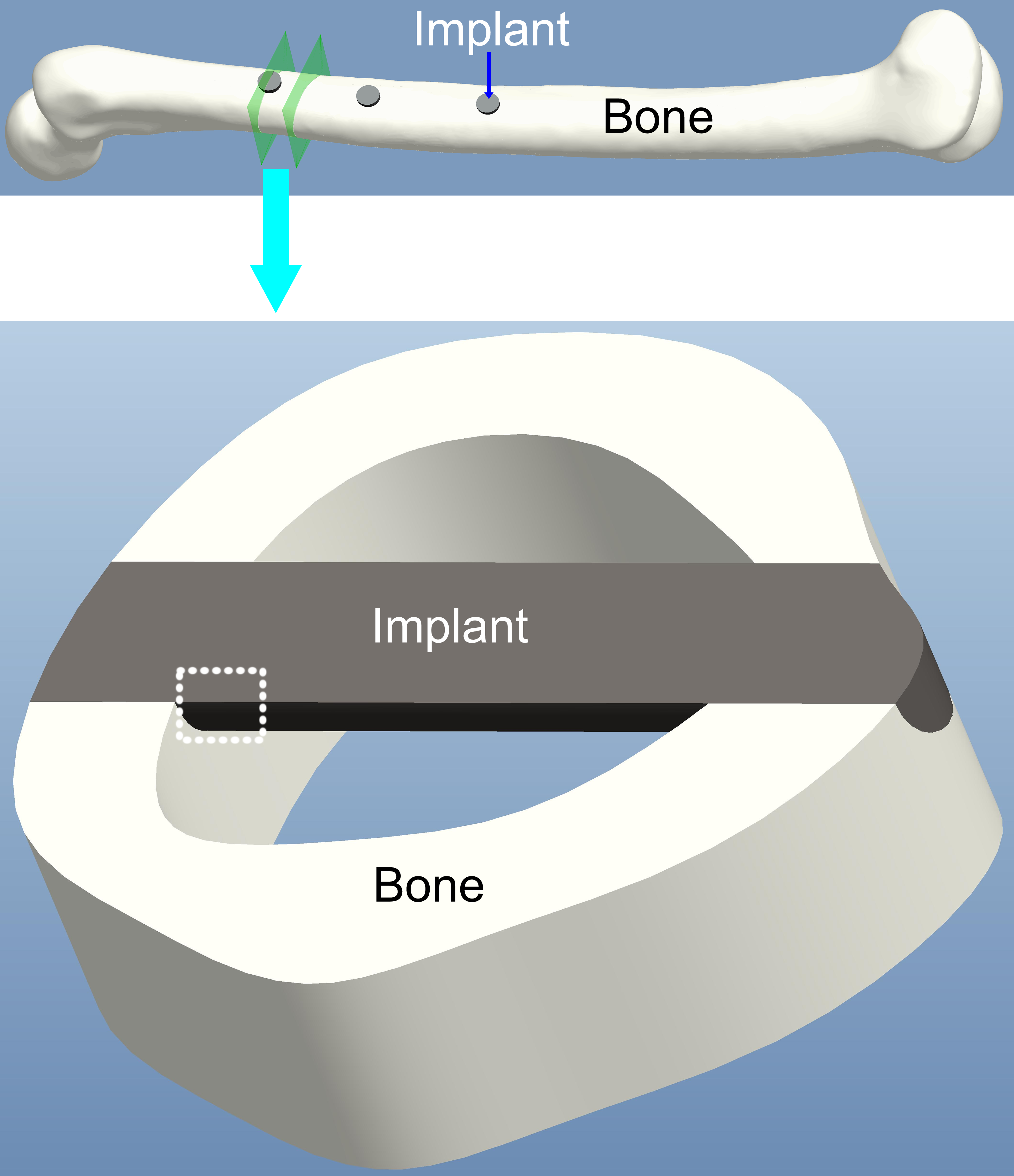
**

**Figure S2.** Schematics showing the position of implant in the rabbit femoral shaft. The square indicates the region for histological analysis.
